# Proarrhythmic changes in human cardiomyocytes during hypothermia by milrinone and isoprenaline, but not levosimendan: an experimental in vitro study

**DOI:** 10.1186/s13049-023-01134-5

**Published:** 2023-10-25

**Authors:** Anders Lund Selli, Mohammadreza Ghasemi, Taylor Watters, Francis Burton, Godfrey Smith, Erik Sveberg Dietrichs

**Affiliations:** 1 Experimental and Clinical Pharmacology, Department of Medical Biology, Faculty of Health Sciences, UiT – The Arctic University of Norway, Postboks 6050, 9037 Langnes, Tromsø, Norway; 2 Center for Psychopharmacology, Diakonhjemmet Hospital, Oslo, Norway; 3 Institute of Oral Biology, University of Oslo, Oslo, Norway; 4 Institute of Cardiovascular and Medical Sciences, University of Glasgow, Glasgow, Scotland; 5 Clyde Biosciences, Newhouse, Scotland

**Keywords:** Levosimendan, Milrinone, Isoprenaline, Hypothermia, Cardiomyocytes, Electrophysiology, Contractility, Accidental hypothermia

## Abstract

**Background:**

Accidental hypothermia, recognized by core temperature below 35 °C, is a lethal condition with a mortality rate up to 25%. Hypothermia-induced cardiac dysfunction causing increased total peripheral resistance and reduced cardiac output contributes to the high mortality rate in this patient group. Recent studies, in vivo and in vitro, have suggested levosimendan, milrinone and isoprenaline as inotropic treatment strategies in this patient group. However, these drugs may pose increased risk of ventricular arrhythmias during hypothermia. Our aim was therefore to describe the effects of levosimendan, milrinone and isoprenaline on the action potential in human cardiomyocytes during hypothermia.

**Methods:**

Using an experimental in vitro-design, levosimendan, milrinone and isoprenaline were incubated with iCell^2^ hiPSC-derived cardiomyocytes and cellular action potential waveforms and contraction were recorded from monolayers of cultured cells. Experiments were conducted at temperatures from 37 °C down to 26 °C. One-way repeated measures ANOVA was performed to evaluate differences from baseline recordings and one-way ANOVA was performed to evaluate differences between drugs, untreated control and between drug concentrations at the specific temperatures.

**Results:**

Milrinone and isoprenaline both significantly increases action potential triangulation during hypothermia, and thereby the risk of ventricular arrhythmias. Levosimendan, however, does not increase triangulation and the contractile properties also remain preserved during hypothermia down to 26 °C.

**Conclusions:**

Levosimendan remains a promising candidate drug for inotropic treatment of hypothermic patients as it possesses ability to treat hypothermia-induced cardiac dysfunction and no increased risk of ventricular arrhythmias is detected. Milrinone and isoprenaline, on the other hand, appears more dangerous in the hypothermic setting.

## Background

Accidental hypothermia, a clinical condition recognized as an involuntary drop in core temperature to below 35 °C, is associated with several lethal complications [1] such as ventricular fibrillation (VF) and cardiac arrest [2]. Occurrence of hypothermia-induced ventricular fibrillation was recently suggested to be most probable in moderate hypothermia, at core temperatures around 30 °C [3]. Hypothermia-induced cardiac dysfunction (HCD) is also a clinical challenge in this patient group, characterized by increased total peripheral resistance (TPR) alongside a decreased cardiac output (CO), leading to poor perfusion of the brain and internal organs [4]. This, in turn, leads to a high mortality in hypothermic patients, estimated as high as 25% [5].

Despite the high mortality rate, few studies have been conducted to find pharmacological strategies to reduce and treat the lethal complications of hypothermia. Thus, treatment guidelines for accidental hypothermia recommend withholding any drug administration until a core temperature of at least 30 °C is reached [2]. However, recent studies have shown that phosphodiesterase 3 (PDE3)-inhibitors and β-receptor stimulators have the potential to alleviate HCD in vivo [6–8], and that the cellular mechanisms of these drugs remain intact at temperatures down to 20 °C [9, 10].

The aforementioned drugs do however have potential electrophysiological side effects and may increase the already high risk of ventricular fibrillation in hypothermic patients. Both PDE-inhibitors and β-receptor stimulators increase intracellular levels of cAMP. This could affect the plateau (phase 2) and rapid repolarization (phase 3) of the cardiac action potential (AP), where voltage gated calcium channels normally open and close. Altered intracellular calcium homeostasis could have electrophysiological consequences, leading to afterdepolarizations and ventricular arrhythmias even during normothermia [11]. Our hypothesis was that inotropic drugs elevating cAMP could increase risk for arrhythmias in hypothermic conditions. Since evidence of such temperature-dependent electrophysiological side effects are lacking, we aimed to explore the effects of PDE3-inhibitors levosimendan and milrinone, as wells as the β-receptor stimulator isoprenaline, on AP duration and contractility of human cardiomyocytes during different stages of hypothermia. Human-induced pluripotent stem-cell derived cardiomyocytes (hiPSC-CM) have previously been demonstrated to be a reliable method to detect pro-arrhythmic effects of drugs during normothermia [12]. We therefore wanted to use this established method during hypothermia, to detect drug induced changes in early and late repolarisation, as measured by time to 30% (APD30) and 90% (APD90) of the total AP duration, as well as triangulation of the AP.

Triangulation is an established marker of cardiac instability and increased arrhythmia risk [12, 22]. It can be calculated by (APD90-APD30)/APD90, and thus gives an estimate on relative repolarization in phase 2 and 3 of the cardiac AP. Such prolongation of phase 2 and 3 is the substrate for *Torsades de Pointes* (TdP), which may cause VF and cardiac arrest [23]. It is thought to be the underlying cause of VF during hypothermia, where decreased QRS/QTc-ratio has been suggested as a superior ECG-marker over QTc for increased VF-risk [15]. The underlying rationale is that QRS/QTc indicates prolonged repolarization relative to conduction time and ventricular depolarization. Accordingly, both QRS/QTc and triangulation of the cardiac AP are more powerful tools to evaluate changes in phase 2 and 3 repolarization than QTc or APD90 alone. In addition to measure the arrhythmogenic effects of these relevant drugs for inotropic support, the hiPSC-CM model allows us to do simultaneous contractility measurements and acquire important information for future improvement of treatment strategies in victims of accidental hypothermia.

## Methods

**Fig. 1:**
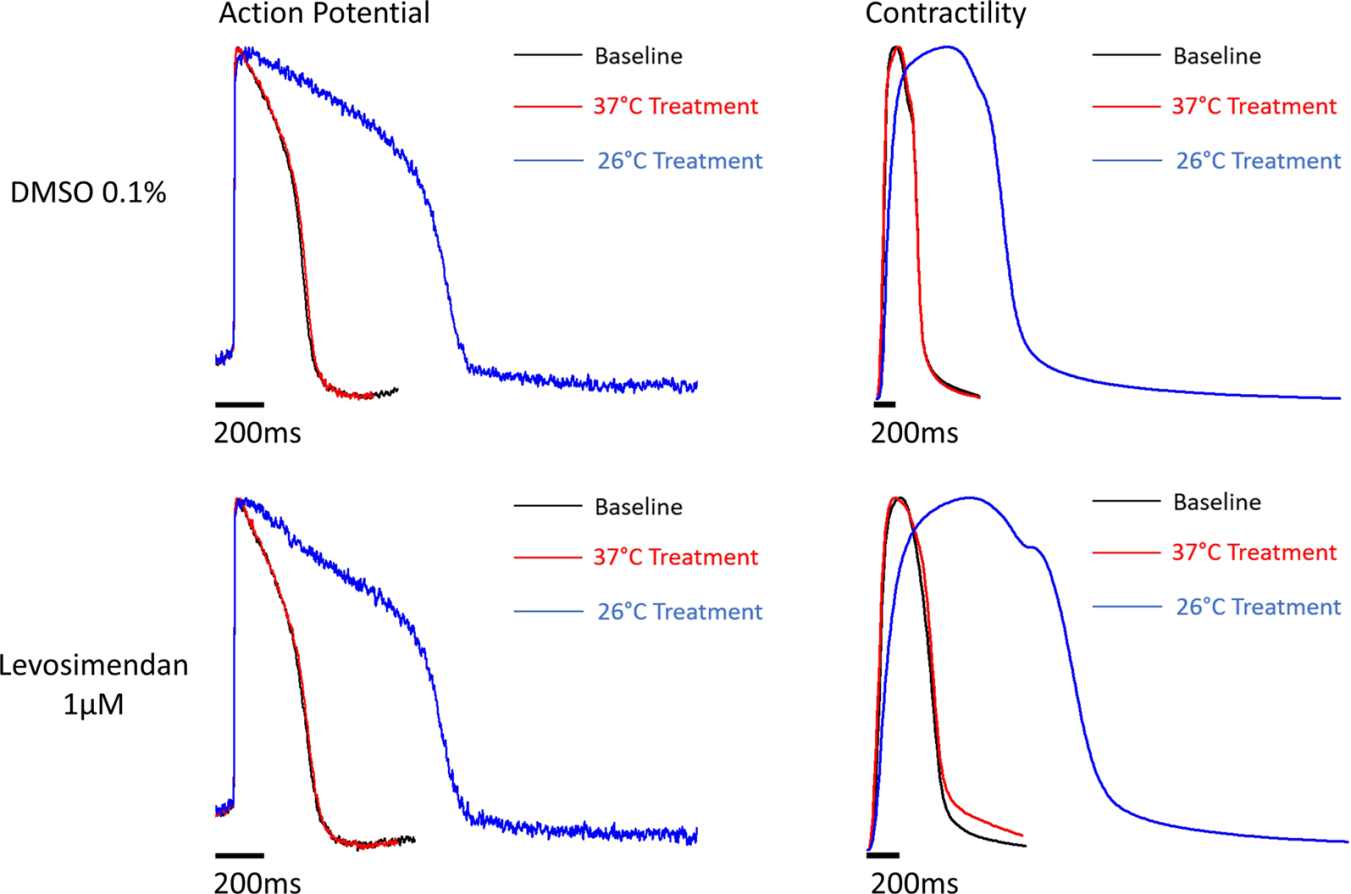
Average action potentials and contraction recordings from DMSO 0.1% treated wells (top) and levosimendan 1 µM treated wells (bottom) at baseline, normothermia (37 °C) and severe hypothermia (26 °C)

The effects of levosimendan (U. S. Pharmacopeia, Rockville, MD, USA) at 0.1, 1 and 10 µM, milrinone (Sigma-Aldrich, Steinheim, Germany) at 1, 10 and 100 µM and isoprenaline (Sigma-Aldrich, Steinheim, Germany) at 0.01, 0.1 and 1 µM concentrations, on cellular APs and contraction during hypothermia was recorded in a hiPSC-CM model, using iCell^2^ hiPSC-derived cardiomyocytes (FujiFilm CDI, Madison, WI, USA). This was performed using a voltage-sensitive dye system (CellOPTIQ® platform) to determine AP duration (30%) (APD30), AP duration (90%) (APD90), triangulation of APs ((APD90-APD30)/APD90), amplitude of contractions (CtAmp), contractility duration at 50% amplitude (CtD50) and contraction rise time (CtTrise) for each individual well treated with test drugs or control (Fig. 1).

### Experimental protocol

1000× stock solutions of the test item was made in dimethyl sulfoxide (DMSO). The 1000× stock solutions was diluted in serum-free media to make 2× solutions. To apply the test item to the cells, 50% of the media was removed and replaced with an equivalent volume of the 2× solution to arrive at the test concentration with a final DMSO concentration of 0.1%. DMSO (0.1% in serum-free media) was the negative control. Cryopreserved iCell^2^® hiPSC-derived cardiomyocytes (Cellular Dynamic International) was plated using iCell^2^ Cardiomyocyte Plating Medium and maintained in fibronectin coated 96-well plates in iCell^2^ Cardiomyocyte Maintenance Medium (serum containing) provided by the manufacturer. The plated cells were stored in an incubator at 37 °C for at least 5 days with media changes every other day before starting experiments.

iCell^2^ maintenance® media was replaced with fresh serum-free media containing the voltage sensitive dye (1:2000 FluoVolt™, ThermoFisher Scientific) and the plate was placed in an incubator at 37 °C. After a 25-min incubation period, the dye-containing media was replaced with fresh serum-free media. The cells were allowed to equilibrate in serum-free media in an incubator at 37 °C for at least 30 min before starting experiments. The 96-well plate was transferred to CellOPTIQ® platform equipped with a CO_2_ and temperature stage incubator, which allowed the experiment to start in a water saturated atmosphere at 37 °C with 5% CO_2_.

Baseline spontaneous electrical activity was recorded for 10 s from a 0.2 × 0.2 mm area of each well. Fluorescence signals were recorded using a 40X (NA 0.6) objective lens with an excitation wavelength of 470 nm. Emitted light was measured using a single photomultiplier tube (PMT) and digitized at 10 kHz. Baseline videos (100 Hz frame rate) for cellular contractility characterization was recorded for 20 s from the same 0.2 × 0.2 mm area of each well using white light.

After recording the baseline behaviour, 50% of the media in each well was replaced with 2× the final test item concentration or control solutions. Three concentrations of the test item were tested in 9 wells each, along with 15 wells for negative control (0.1% DMSO). Contractile and electrical activity was recorded from the same area within each well that was selected for baseline characterization. The temperature of the stage incubator was reduced to 34 °C and allowed to equilibrate. Following equilibration contractile and electrical activity was recorded from the same area within each well that was selected for baseline characterization. This step was repeated for measurements at 30 °C, 26 °C and rewarming to 37 °C. The last recording was after 24 h at 37 °C.

### Ethics statement:

The study was conducted on commercially available cardiomyocytes derived from pluripotent stem cells, and no approval from either Norwegian or Scottish health authorities were needed.

### Statistics

Repeated measures one-way ANOVA with Holm-Sidak was used to evaluate differences from baseline for DMSO 0.1% and all concentrations of all three drugs. When data was not normally distributed, repeated measures one-way ANOVA repeated measures with Dunn`s post hoc test was performed. One-way ANOVA with Holm-Sidak was performed to evaluate differences from negative control (DMSO 0.1%) for each drug at each temperature. When data was not normally distributed, one-way ANOVA on ranks with Dunn`s test post hoc was performed. To evaluate differences between concentrations for each drug at each temperature, one-way ANOVA with Holm-Sidak post hoc test was performed. When data was not normally distributed, ANOVA on ranks was performed with Tukey post hoc test. Differences between groups were considered significant when *p* values were < 0.05. All analysis were performed using SigmaPlot 14.5 (Systat Software, San Jose, CA, USA). Figures were created with SigmaPlot 14.5 (Systat Software, San Jose, CA, USA). All data are presented as mean ± standard error of mean (S.E.M.).

### Assessment of Q_10_

Q_10_ is a method of assessing the temperature sensitivity of a biological process [13]. The rate of repolarization was estimated by 1/APD90 (ms^−1^) on the basis that the AP amplitude was similar regardless of temperature and therefore the inverse of the APD90 reflects the summed rates of the ionic processes that are responsible for repolarization.

Evaluation of translational potential for our data was performed by comparing Q_10_-values based on the APD90 and rate-corrected APD90 values to the Q_10_-values based on QT and QTc from reported data on human electrophysiology during hypothermia, obtained through a structured PubMed-search. Included papers had to report human adults and report absolute QT or QTc. No case reports were included.

Q_10_ was calculated as (R2/R1)^10/(T2–T1), where R2 and R1 are 1/APD90 for temperatures T2 and T1 respectively. For the human ECG-data R2 and R1 were 1/QT or 1/QTc recorded from ECGs. Q_10_ for HR of the included papers were also calculated and compared to Q_10_ values based on the measurements of the spontaneous rate of hi-PSC CMs.

### Role of funders

The funders of this study did not have any role in study design, data collection, data analyses, interpretation, or writing of report.

## Results

### Electrophysiological data:

Except for the AP upstroke (Ct Rise) and triangulation of the AP, hypothermia had a clear temperature-dependent effect on electrophysiology, by markedly increasing the duration of measured AP parameters.

#### Effects on APD30 (Fig. 2)

**Fig. 2:**
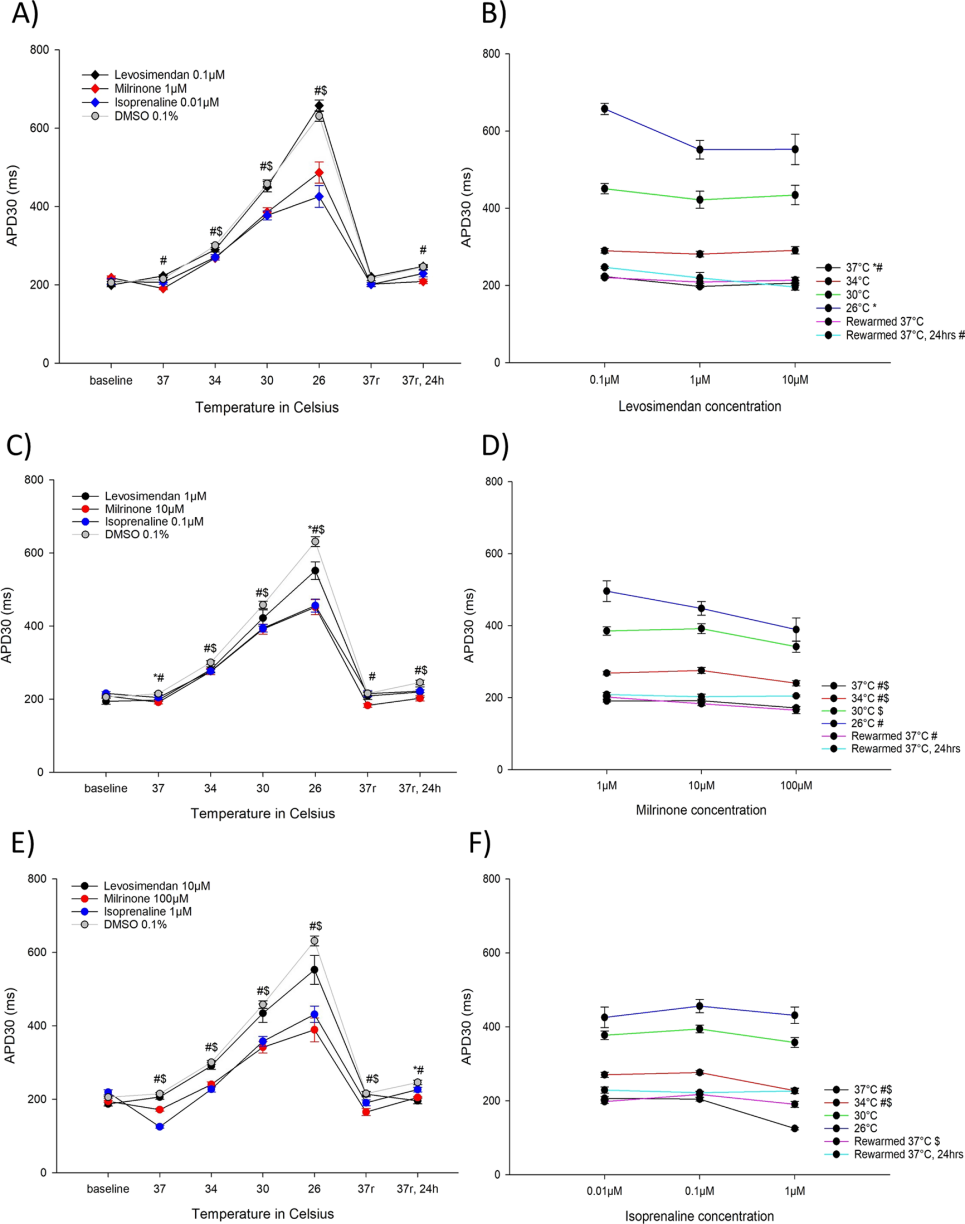
A APD30 (ms) for the lowest concentrations of levosimendan (0.1 µM, n = 9), milrinone (1 µM, n = 9) and isoprenaline (0.01 µM, n = 9) during hypothermia and rewarming. B APD30 (ms) for the different levosimendan concentrations (0.1 µM, 1 µM and 10 µM). C APD30 (ms) for the intermediate concentrations of levosimendan (1 µM, n = 9), milrinone (10 µM, n = 9) and isoprenaline (0.1 µM, n = 9) during hypothermia and rewarming. D APD30 (ms) for the different milrinone concentrations (1 µM, 10 µM and 100 µM). E APD30 (ms) for the highest concentrations of levosimendan (10 µM, n = 9), milrinone (100 µM, n = 9) and isoprenaline (1 µM, n = 9) during hypothermia and rewarming. F APD30 (ms) for the different isoprenaline concentrations (0.01 µM, 0.1 µM and 1 µM). A, C, E *Significant differences between levosimendan and DMSO 0.1% (n = 15), ^#^significant difference between milrinone and DMSO 0.1% (n = 15), ^$^significant difference between isoprenaline and DMSO 0.1% (n = 15). B, D, F *Significant difference between lowest and intermediate concentration, ^#^significant difference between lowest and highest concentration, ^$^significant difference between intermediate and highest concentration

Levosimendan 1 µm decreased APD30 compared to untreated control at 37 °C and 26 °C, while levosimendan 10 µM decreased APD30 24 h after rewarming to 37 °C. Except from 1 µM milrinone after rewarming to 37 °C, all milrinone concentrations decreased APD30 at all temperatures of the protocol. At 34 °C, 30 °C and 26 °C all three concentrations of isoprenaline shortened APD30. 24 h after rewarming to 37 °C, 0.1 µM isoprenaline decreased APD30. The same applied for 1 µM isoprenaline at 37 °C and after rewarming.

#### Effects on APD90 (Fig. 3)

**Fig. 3:**
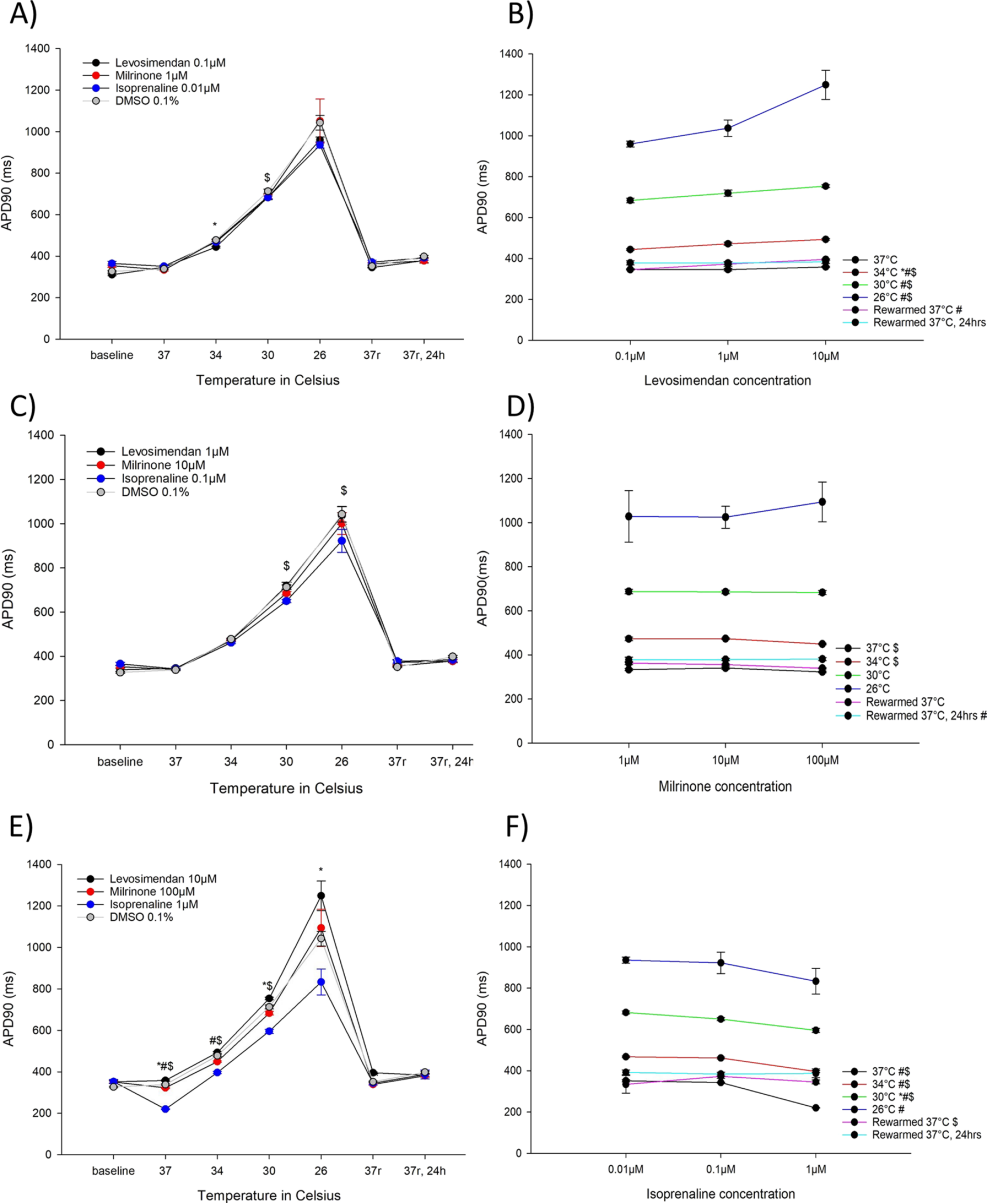
A APD90 (ms) for the lowest concentrations of levosimendan (0.1 µM, n = 9), milrinone (1 µM, n = 9) and isoprenaline (0.01 µM, n = 9) during hypothermia and rewarming. B APD90 (ms) for the different levosimendan concentrations (0.1 µM, 1 µM and 10 µM). C APD90 (ms) for the intermediate concentrations of levosimendan (1 µM, n = 9), milrinone (10 µM, n = 9) and isoprenaline (0.1 µM, n = 9) during hypothermia and rewarming. D APD90 (ms) for the different milrinone concentrations (1 µM, 10 µM and 100 µM). E APD90 (ms) for the highest concentrations of levosimendan (10 µM, n = 9), milrinone (100 µM, n = 9) and isoprenaline (1 µM, n = 9) during hypothermia and rewarming. F APD90 (ms) for the different isoprenaline concentrations (0.01 µM, 0.1 µM and 1 µM). A, C, E *Significant differences between levosimendan and DMSO 0.1% (n = 15), ^#^significant difference between milrinone and DMSO 0.1% (n = 15), ^$^significant difference between isoprenaline and DMSO 0.1% (n = 15). B, D, F *Significant difference between lowest and intermediate concentration, ^#^significant difference between lowest and highest concentration, ^$^significant difference between intermediate and highest concentration

Levosimendan 10 µM increased APD90 at 37 °C, 30 °C, 26 °C and after rewarming to 37 °C. Levosimendan 0.1 µM shortened APD90 at 34 °C. 100 µM milrinone decreased APD90 at 37 °C and 34 °C. 1 µM isoprenaline shortened APD90 at 37 °C, 34 °C, 30 °C and 26 °C. At 30 °C and 26 °C, 0.1 µM isoprenaline also decreased APD90. 0.01 µM isoprenaline shortened APD90 at 30 °C.

#### Effects on AP triangulation (Fig. 4)

**Fig. 4:**
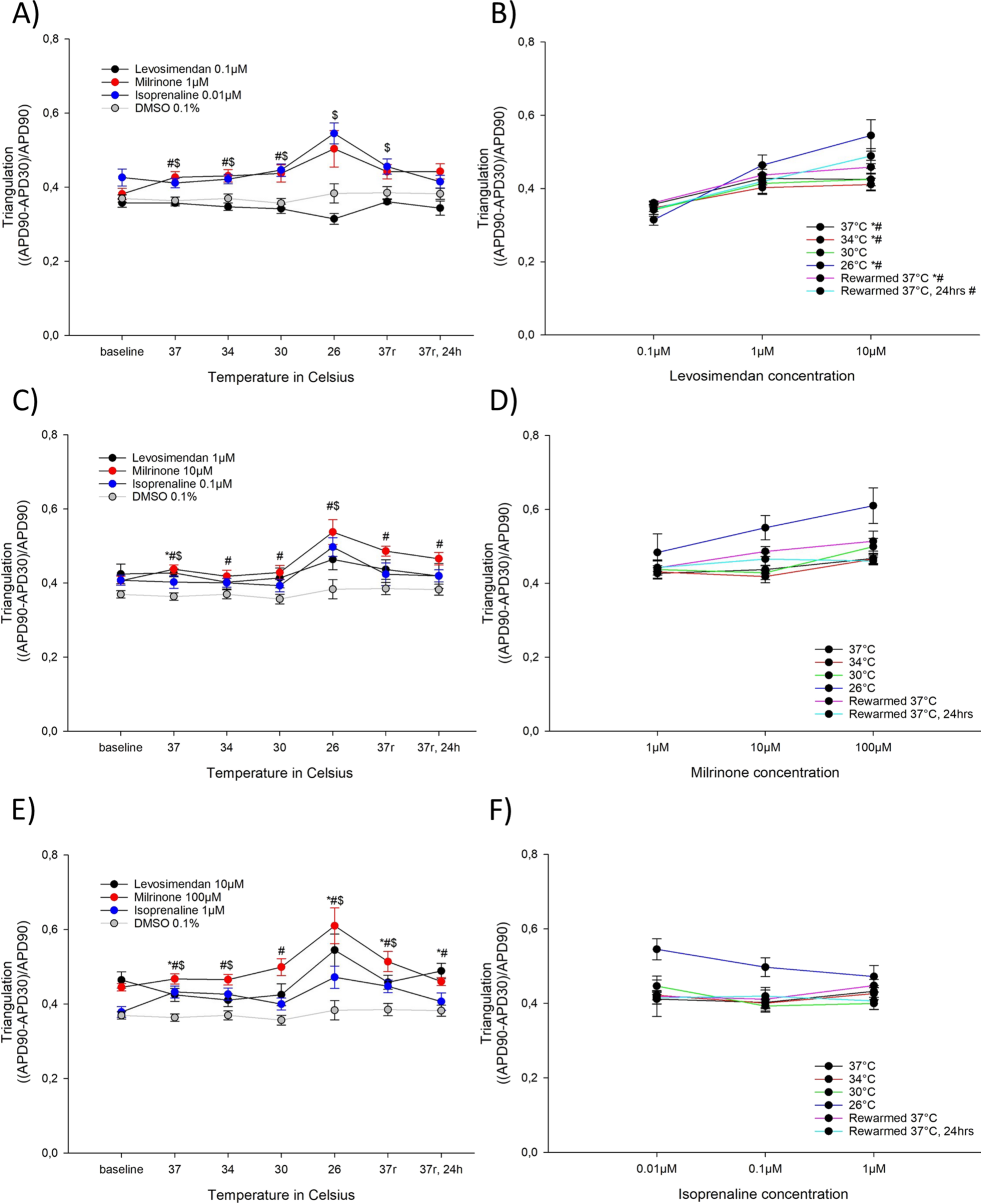
A Triangulation ((APD90-APD30)/APD90) for the lowest concentrations of levosimendan (0.1 µM, n = 9), milrinone (1 µM, n = 9) and isoprenaline (0.01 µM, n = 9) during hypothermia and rewarming. B Triangulation ((APD90-APD30)/APD90) for the different levosimendan concentrations (0.1 µM, 1 µM and 10 µM). C Triangulation ((APD90-APD30)/APD90) for the intermediate concentrations of levosimendan (1 µM, n = 9), milrinone (10 µM, n = 9) and isoprenaline (0.1 µM, n = 9) during hypothermia and rewarming. D Triangulation ((APD90-APD30)/APD90) for the different milrinone concentrations (1 µM, 10 µM and 100 µM). E Triangulation ((APD90-APD30)/APD90) for the highest concentrations of levosimendan (10 µM, n = 9), milrinone (100 µM, n = 9) and isoprenaline (1 µM, n = 9) during hypothermia and rewarming. F Triangulation ((APD90-APD30)/APD90) for the different isoprenaline concentrations (0.01 µM, 0.1 µM and 1 µM). A, C, E *Significant differences between levosimendan and DMSO 0.1% (n = 15), ^#^significant difference between milrinone and DMSO 0.1% (n = 15), $significant difference between isoprenaline and DMSO 0.1% (n = 15). B, D, F *Significant difference between lowest and intermediate concentration, ^#^significant difference between lowest and highest concentration, ^$^significant difference between intermediate and highest concentration

10 µM levosimendan caused an increase in triangulation at 37 °C, 26 °C, after rewarming to 37 °C and 24 h after rewarming to 37 °C. 1 µM levosimendan increased triangulation at 37 °C. The two highest concentration of milrinone, 100 µM and 10 µM, increased triangulation throughout the temperature protocol. At 37 °C, 34 °C and 30 °C, 1 µM milrinone also prolonged triangulation more than untreated control. At 37 °C and 26 °C, all three concentrations of isoprenaline prolonged triangulation. At 34 °C 0.01 µM and 1 µM increased triangulation, while at 30 °C and after rewarming to 37 °C 0.01 µM isoprenaline prolonged triangulation. After rewarming to 37 °C, 1 µM also prolonged triangulation.

**Fig. 5:**
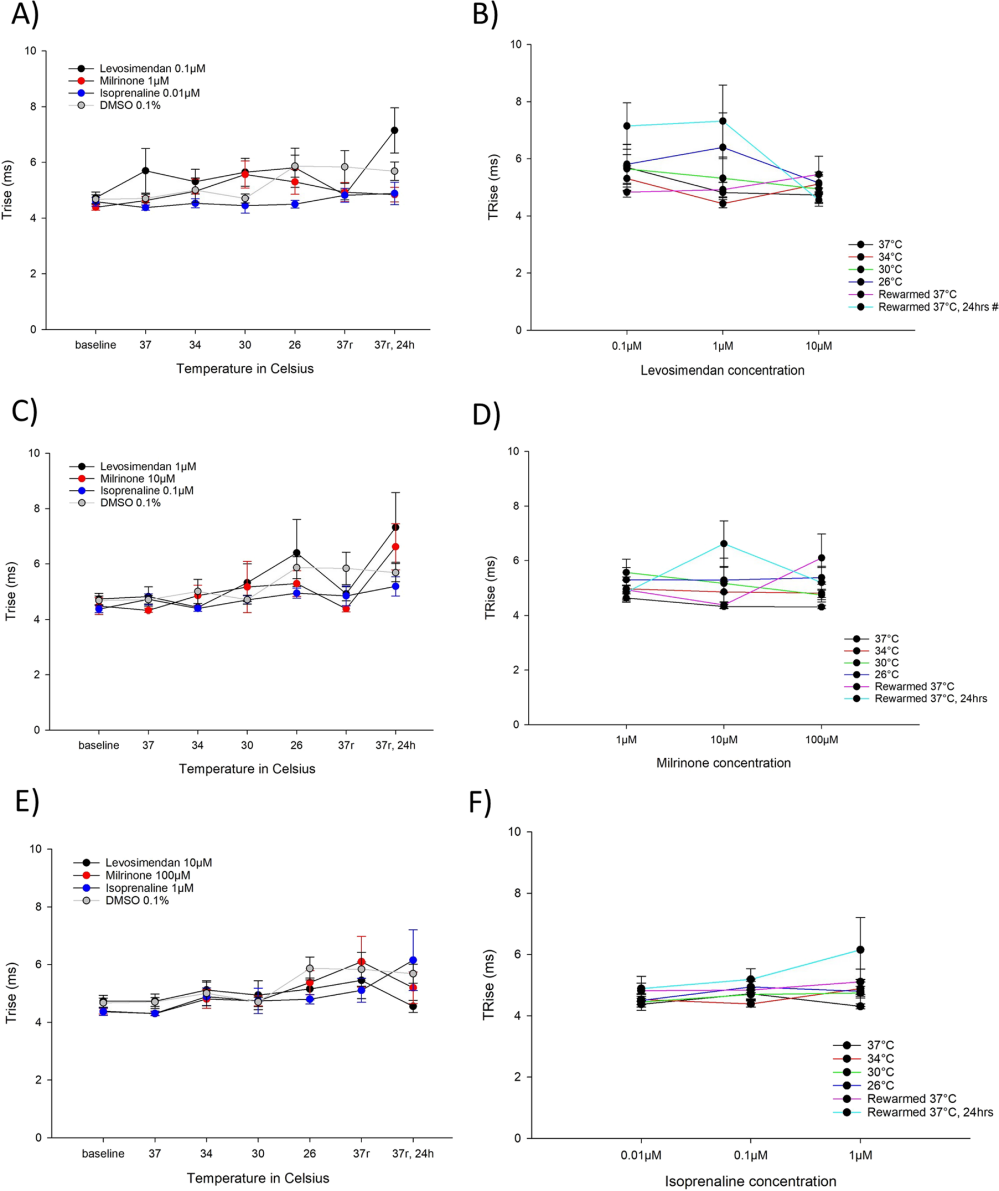
**A** Upstroke of action potential, Trise (ms), for the lowest concentrations of levosimendan (0.1 µM, n = 9), milrinone (1 µM, n = 9) and isoprenaline (0.01 µM, n = 9) during hypothermia and rewarming. **B** Upstroke of action potential, Trise (ms), for the different levosimendan concentrations (0.1 µM, 1 µM and 10 µM). **C** Upstroke of action potential, Trise (ms), for the intermediate concentrations of levosimendan (1 µM, n = 9), milrinone (10 µM, n = 9) and isoprenaline (0.1 µM, n = 9) during hypothermia and rewarming. **D **Upstroke of action potential, Trise (ms), for the different milrinone concentrations (1 µM, 10 µM and 100 µM). **E** Upstroke of action potential, Trise (ms), for the highest concentrations of levosimendan (10 µM, n = 9), milrinone (100 µM, n = 9) and isoprenaline (1 µM, n = 9) during hypothermia and rewarming. **F** Upstroke of action potential, Trise (ms), for the different isoprenaline concentrations (0.01 µM, 0.1 µM and 1 µM). **A, C, E** *Significant differences between levosimendan and DMSO 0.1% (n = 15), ^#^significant difference between milrinone and DMSO 0.1% (n = 15), $significant difference between isoprenaline and DMSO 0.1% (n = 15). **B, D, F** *Significant difference between lowest and intermediate concentration, ^#^significant difference between lowest and highest concentration, ^$^significant difference between intermediate and highest concentration

Effects on AP upstroke (Fig. 5): None of the drugs, at any concentration, caused a change in the AP upstroke (Trise) compared to untreated control at any point of the protocol.

### Contractility data

Except for the contraction amplitude, hypothermia had a clear temperature-dependent effect on measured contractility data, by markedly increasing the duration-time of contractions and contraction rise time.

#### Effects on contraction duration (Fig. 6)

**Fig. 6:**
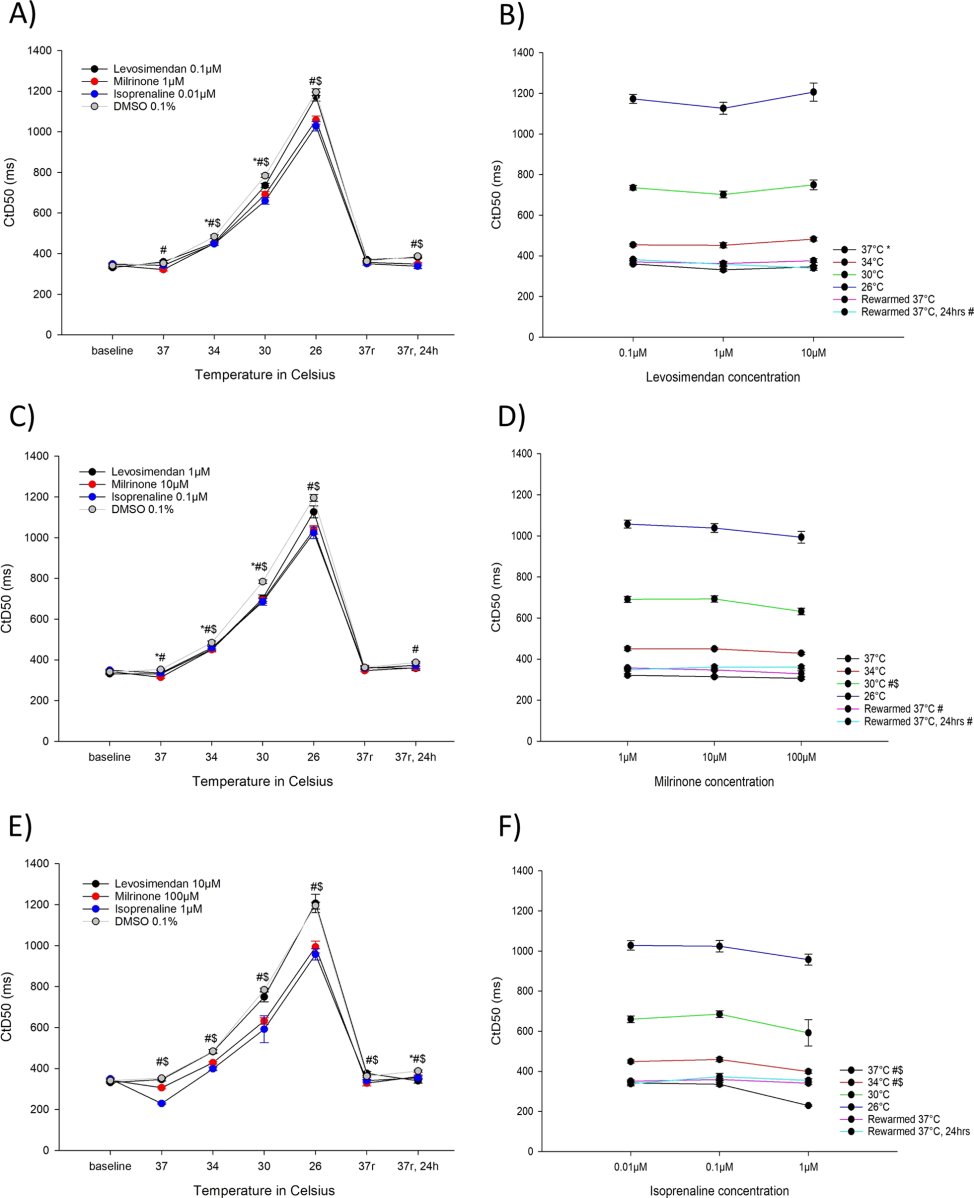
A Contraction duration, CtD50 (ms), for the lowest concentrations of levosimendan (0.1 µM, n = 9), milrinone (1 µM, n = 9) and isoprenaline (0.01 µM, n = 9) during hypothermia and rewarming. B Contraction duration, CtD50 (ms), for the different levosimendan concentrations (0.1 µM, 1 µM and 10 µM). C Contraction duration, CtD50 (ms), for the intermediate concentrations of levosimendan (1 µM, n = 9), milrinone (10 µM, n = 9) and isoprenaline (0.1 µM, n = 9) during hypothermia and rewarming. D Contraction duration, CtD50 (ms), for the different milrinone concentrations (1 µM, 10 µM and 100 µM). E Contraction duration, CtD50 (ms), for the highest concentrations of levosimendan (10 µM, n = 9), milrinone (100 µM, n = 9) and isoprenaline (1 µM, n = 9) during hypothermia and rewarming. F Contraction duration, CtD50 (ms), for the different isoprenaline concentrations (0.01 µM, 0.1 µM and 1 µM). A, C, E *Significant differences between levosimendan and DMSO 0.1% (n = 15), ^#^significant difference between milrinone and DMSO 0.1% (n = 15), $significant difference between isoprenaline and DMSO 0.1% (n = 15). B, D, F *Significant difference between lowest and intermediate concentration, ^#^significant difference between lowest and highest concentration, ^$^significant difference between intermediate and highest concentration

0.1 µM levosimendan decreased CtD50 compared to untreated control at 34 °C and 30 °C. A decrease in CtD50 was also caused by 1 µM levosimendan at 37 °C, 34 °C and 30 °C. After 24 h of rewarming at 37 °C, 10 µM levosimendan decreased CtD50. All three concentrations of milrinone decreased CtD50 compared to untreated control at all temperatures except after rewarming to 37 °C, where only 100 µM milrinone gave a decrease. At 34 °C, 30 °C and 26 °C, all concentrations of isoprenaline decreased CtD50 compared to untreated control. 1 µM isoprenaline decreased CtD50 throughout our protocol. 0.01 µM isoprenaline decreased CtD50 compared to untreated control after 24 h of rewarming at 37 °C.

#### Effects on rate of rise of contraction (Fig. 7)

**Fig. 7:**
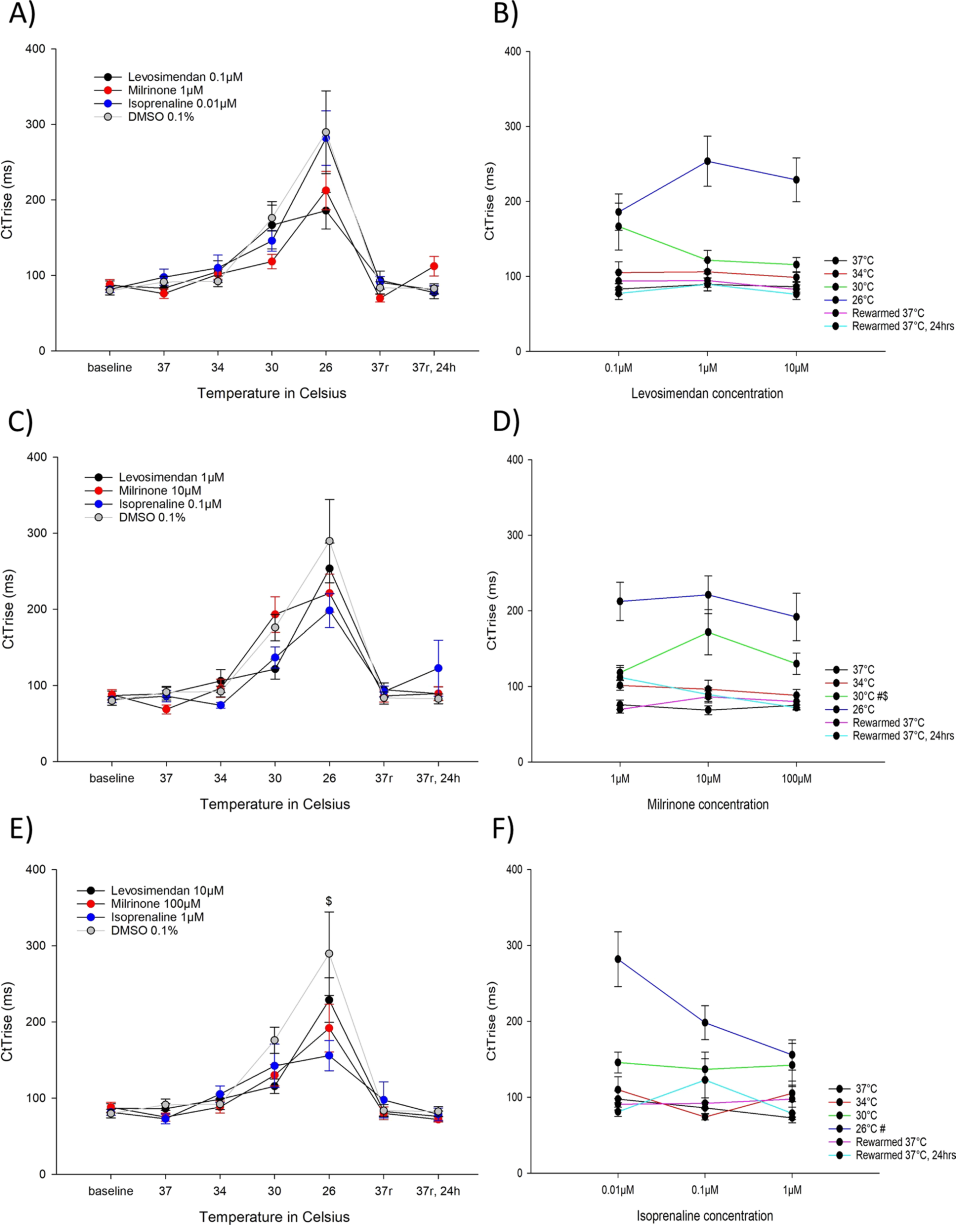
A Contraction rise time, CtTrise (ms), for the lowest concentrations of levosimendan (0.1 µM, n = 9), milrinone (1 µM, n = 9) and isoprenaline (0.01 µM, n = 9) during hypothermia and rewarming. B Contraction rise time, CtTrise (ms), for the different levosimendan concentrations (0.1 µM, 1 µM and 10 µM). C Contraction rise time, CtTrise (ms), for the intermediate concentrations of levosimendan (1 µM, n = 9), milrinone (10 µM, n = 9) and isoprenaline (0.1 µM, n = 9) during hypothermia and rewarming. D Contraction rise time, CtTrise (ms), for the different milrinone concentrations (1 µM, 10 µM and 100 µM). E Contraction rise time, CtTrise (ms), for the highest concentrations of levosimendan (10 µM, n = 9), milrinone (100 µM, n = 9) and isoprenaline (1 µM, n = 9) during hypothermia and rewarming. F Contraction rise time, CtTrise (ms), for the different isoprenaline concentrations (0.01 µM, 0.1 µM and 1 µM). A, C, E *Significant differences between levosimendan and DMSO 0.1% (n = 15), ^#^significant difference between milrinone and DMSO 0.1% (n = 15), $significant difference between isoprenaline and DMSO 0.1% (n = 15). B, D, F *Significant difference between lowest and intermediate concentration, ^#^significant difference between lowest and highest concentration, ^$^significant difference between intermediate and highest concentration

No levosimendan or milrinone concentrations showed alternations in CtTrise compared to untreated control at any temperature. At 26 °C, 1 µM isoprenaline decreased CtTrise compared to untreated control.

#### Effects on contraction amplitude

Levosimendan did not affect the contraction amplitude. After rewarming to 37 °C, 10 µM milrinone increased CtAmp compared to untreated control, while 100 µM milrinone decreased CtAmp compared to untreated control 24 h after rewarming at 37 °C. Both 0.1 µM and 1 µM of isoprenaline increased CtAmp after rewarming to 37 °C.

### Q_10_ values for APD90 and QT (Tables 1, 2)

Q_10_ for the APD90 values reported in this study with reference to 37 °C, at 34 °C, 30 °C and 26 °C were 3.2, 2.9 and 2.8, respectively. After correction for changes in spontaneous rate [14] the Q_10_ values were 2.3 for all 3 temperature ranges. 7 papers reporting QT-times met our criteria for comparison, and one additional paper was included after screening references [15–21]. Q_10_ calculated with 37 °C and various temperatures for therapeutic hypothermia (35–32 °C) for human data showed Q_10_-values ranging from 1.1 to 2.4.

Q_10_ values for the changes in spontaneous rate of the hi-PSC CMs for the 3 temperatures tested ranged from 3.5 to 3.9. In vivo results from human studies varied between 1.6 to 3.4 in the four papers that included data on heart rate.

**Table 1:** Q10-values for QT and QTc during mild (35–32 °C), moderate (32–28 °C) and severe (< 28 °C) hypothermia

Q10-values	hi-PSC-CM		Dietrichs et al	Thomsen et al		Yoon et al		Rosol et al		Salinas et al		Weitz et al		Kim et al	
Parameter	APD90	APD90c	QTc	QT	QTc	QT	QTc	QT	QTc	QT	QTc	QT	QTc	QT	QTc
Mild hypothermia	3.2	2.3	1.4	1.4	1.1	2.4	1.4	2.0	1.3	1.6–2.2	1.3–1.5	2.1	1.6	2.1	1.3
Moderate hypothermia	2.9	2.3	1.2												
Severe hypothermia	2.8	2.3	1.2												

Data was derived from a literature search in PubMed for (therapeutic hypothermia) and (QT interval) and a search for (deep hypothermia) and (QT interval). Included papers had to report human, adult data from the last 10 years and report absolute QT or QTc. The search for deep hypothermia did not have to include data from the last 10 years. No case reports were included. Reference lists of included papers were screened for relevant papers. Thomsen et al. are estimated Q_10_-value by deriving approximate numbers from graphs

**Table 2:** Q10-values for heart rate (in beats per minute, BPM) derived from included papers from literature search compared to beats per minute (BPM) calculated from the equation 1000/cycle length × 60 for hi-PSC CMs

Q10-values	hi-PSC-CM	Yoon et al	Salinas et al	Weitz et al	Kim et al
Parameter	BPM	BPM	BPM	BPM	BPM
Mild hypothermia	3.5	3.3	1.6–2.1	3.4	2.1
Moderate hypothermia	3.7				
Severe hypothermia	3.9				

## Discussion

This experimental study suggests that cardiovascular support during hypothermia with levosimendan should be preferred over isoprenaline and milrinone, as the relative risk for developing ventricular arrhythmias, based on the electrophysiological changes of hiPSC-CMs, is lower. In contrast to levosimendan (Fig. 1), both milrinone and isoprenaline increased AP triangulation for all concentrations and may therefore be less safe in a hypothermic setting. As all three drugs have been suggested as treatment options for hypothermic patients, this observation adds valuable information on safety issues with milrinone and isoprenaline and indicates that levosimendan may still be considered a promising agent to treat HCD.

The present results are supporting the hypothesis that a heterogenic effect of hypothermia on depolarization/ repolarization is pro-arrhythmic [3, 24, 24], as we show no significant changes in Trise (depolarization) down to 26 °C, whereas APD90 increases by ≈ 300%. Accordingly, both QRS/QTc and triangulation of the cardiac AP are more powerful tools to evaluate changes in phase 2 and 3 repolarization than QTc or APD90 alone. Such careful evaluation of repolarization is evident in the present study, as only small differences compared to untreated control in APD90 are observed for most drug concentrations, which could indicate little effect on repolarization. However, since APD30-decrease is significant for both milrinone and isoprenaline, the phase 2 and 3 prolongation of repolarization is only detectable by triangulation. Levosimendan, in its therapeutic concentration range (0.1–1 µM), has small effects on the APD30 and APD90, and consequently triangulation during hypothermia down to 26 °C. This indicates low risk of ventricular arrhythmias in our study. Our findings are in correspondence with recently published results from Chien et al. [25] stating that levosimendan prevents ventricular arrhythmias during therapeutic hypothermia (30 °C). The authors hypothesized that activation of the ATP-sensitive potassium current IK-ATP by levosimendan during hypothermia explained decreased APD, which in turn reduced VF-risk. As activation of I_K-ATP_ accelerates repolarization, it causes a relative shortening of repolarization to AP duration, which we hypothesize may be the mechanism underlying the low VF-risk in levosimendan-treated rabbit hearts [25] and our hi-PSC-derived cardiomyocytes during hypothermia.

The present study also shows that iCell^2^ hiPSC-derived cardiomyocytes using the CellOPTIQ® platform is a possible approach for testing hypothermic effects of various drugs, as we see clear temperature-dependent variations throughout the protocol consistent with physiological responses to decreased human body temperature [26]. The human iPSC-CM monolayer has become a standard in vitro model of human ventricular electrophysiology for pro-arrhythmic assessment [12] as the AP waveform and the spontaneous rate of these preparations has many features similar to the intact ventricle. To compare the temperature sensitivity of iPSC-CM electrophysiology with that of the intact ventricle, Q_10_ values were calculated for hypothermic conditions (Tables 1, 2). The challenge in evaluating both in vitro and in vivo measurements is the confounding effects of temperature on the spontaneous rate of the cardiac systems, as rate independently affects the APD90 and also the QT interval [27, 28]. After correction for rate, the Q_10_ values from the APD90 were comparable to the Q_10_ values based on in vivo QT values. This suggests that the temperature sensitivity of the underlying ion-channels that are the basis of these changes in both in vitro and in vivo systems are comparable and adds additional validity to the electrophysiology of the human iPSC-CM preparation. The Q_10_ value of iPSC-CM spontaneous rate was higher than most estimates of the Q_10_ of spontaneous human heart rates in hypothermia. This is not unexpected as the in vitro iPSC-CM system lacks the modulation of rate by the autonomic nervous system present in the human heart.

Reasons for the variability between the different human studies may be explained by multimorbidity among the patients recovering for cardiac arrest and various anesthetic protocols during cooling. The reason we wanted to evaluate papers reporting therapeutic hypothermia and deep hypothermia during surgery was to reduce confounding neurohormonal impact by sympathetic activation in trauma patients and exposed persons suffering from accidental hypothermia. Although APD90-values being close to the same range as human data, we found no examples above 3.20 (Q_10_ from the present study), indicating that possible additional effects of in vivo hypothermia on other organ systems than the cardiomyocytes alone, such as the sympathetic/parasympathetic nervous system, must be taken into consideration when evaluating the present data. It is, however, interesting to note that isolated rabbit hearts, also without influence of nervous stimuli, showed Q_10_ for QT-interval at 3.85 for 31 °C and 5.25 for 17 °C [3]. Further, in therapeutic hypothermia at 33 °C on deeply anesthetized healthy pigs, Q_10_-value for QTc was 4.18 and for QT 5.23 [29]. This illustrates that the present model using hi-PSC-CMs during hypothermia provides reliable results on temperature effects on cardiac electrophysiology and may spare animal studies performed for this purpose.

Inotropic effect of all three drugs appears preserved, although with larger data variability, during hypothermia down to 26 °C according to this study. Levosimendan has previously shown ability to alleviate HCD in vivo through inotropic and vasodilatory effects resulting in decreased TPR and increased CO [7, 30]. Its pharmacodynamic effects are intact during cooling to 20 °C [10]. In addition, a large in vivo study including 40 male Wistar rats rewarmed from deep hypothermic circulatory arrest (13–15 °C) showed that levosimendan was superior to epinephrine for treatment of myocardial dysfunction during rewarming [31]. As unstable hypothermia patients are preferably rewarmed using an extra-corporeal membrane oxygenator (ECMO) to achieve sufficient cardiovascular support [2], it is also interesting to note that levosimendan is suggested to improve survival in normothermic patients on ECMO [32, 33]. Our recent results showing that levosimendan does not increase VF-risk during hypothermic conditions, alongside previous findings of positive hemodynamic effects in vivo [31, 34–36], makes levosimendan a very promising pharmacological strategy for cardiovascular support both in accidental and therapeutic hypothermia patients.

β-receptor stimulation through administration of isoprenaline, has earlier been suggested to lack inotropic effects during hypothermia when administered to rats [37, 38]. However, recent studies have shown that β1-stimulus by epinephrine has positive inotropic effects on pigs cooled down to 32 °C [6], which corresponds with the findings in the present study. A case report from 2015 also indicates that isoprenaline had beneficial effect in treatment of VF in an accidental hypothermia patient [39]. The rationale for administering isoprenaline in the described patient was to shorten the prolonged cycle length thought to cause ventricular extrasystoles leading to the VF. Although isoprenaline possess chronotropic effects that could be beneficial in some circumstances, we show elevated risk for isoprenaline-induced VF during hypothermia with increased triangulation. This risk appears most significant for 0.01 µM and 0.1 µM, as 1 µM decreased APD90 and triangulation to a much larger extent. This is in contrast to levosimendan and milrinone which both increase triangulation with increasing concentrations. However, 1 µM isoprenaline is not suitable for clinical use as it extensively exceeds therapeutic concentrations.

We have earlier shown that milrinone possess some of the same positive effects as levosimendan, alleviating HCD in vivo and giving unaltered pharmacodynamic effects in vitro during hypothermia [8, 10]. Others have conflicted these findings and suggested that the positive inotropic effects are suppressed by lowered temperatures, when studying pharmacological effects in guinea pigs [30]. In the present study we show indication for increased VF-risk, predicted by increased triangulation after milrinone exposure during hypothermia. Milrinone should therefore be administered with caution to hypothermic patients.

We show a significant increase in contraction duration (CtD50) compared to normothermia at 34 °C, 30 °C and 26 °C, and CtTrise is increased at 30 °C and 26 °C. As HR decreases with decreasing temperature [27], these are not surprising results. It is, however, interesting to note that although the contraction rise time (CtTrise) increases significantly with hypothermia, the depolarization time (Trise) remains mainly unaltered in the present study. This indicates that the cellular mechanisms of contraction, which are initiated by Ca^2+^-influx [40], are more temperature-sensitive than the Na^2+^-mediated depolarization of the contractile cardiomyocytes. This is supported by a recent systematic review on electrophysiology during hypothermia that summarized that Ca^2+^-influx is increased possibly due to increased APD during cold temperatures, whereas Na^2+^-influx during depolarization possibly remains unaltered [41]. Further, both Trise and CtTrise show no tendency towards differences from untreated control for any of the drugs, whereas contraction duration (CtD50) appears significantly decreased by all concentrations of milrinone and isoprenaline. This could be explained by more prominent cAMP-mediated effects by isoprenaline and milrinone than levosimendan, leading to phosphorylation of phospholamban and cardiac troponin I (cTnI) resulting in accelerated relaxation [42].

More studies on temperature-dependent pharmacokinetics are needed to explore clinical feasibility for the drugs, particularly for levosimendan that shows promising results in this and previous studies. A large review from 2010 concluded that drug specific pharmacokinetics and pharmacodynamics must be determined before clinical implementation is considered [43]. Levosimendan is mainly metabolized through conjugation by glutathione and excreted through urine and faeces, but some of the conjugated levosimendan gets further metabolized to the active metabolite OR-1896 through acetylation [44]. The aforementioned review underlines decreased enzyme activity, altered hepatic and intestinal blood flow, and changed morphology of receptors as some of the potential pharmacotherapeutic challenges during hypothermia [43]. As this may cause increased risk of toxicity and unpredictable side effects with altering temperature, pharmacokinetic evaluation in vivo is needed before clinical use of levosimendan in hypothermic patients. It also remains uncertain how large animals, with larger translational value such as pigs [45], are affected by levosimendan during hypothermia. Continuous monitoring of hemodynamics and ECG, including QRS/QTc-calculation, to detect possible arrhythmias would provide necessary information about the applicability of levosimendan in a hypothermic setting. Such investigations, at temperatures down to 10 °C-15°C would be valuable as these are the lowest temperatures reported in child and adult surviving hypothermia with good neurological outcome [46, 47].

## Conclusion

Levosimendan emerge as a promising drug to treat cardiovascular dysfunction in hypothermic patients as we show no increased risk of ventricular arrhythmias, as well as sustained contractile effects down to 26 °C in human cardiomyocytes. Human pharmacokinetic data of levosimendan during hypothermia are however needed to further evaluate its clinical potential in this setting. Milrinone and isoprenaline both increase triangulation and thereby appears to elevate VF-risk during hypothermia.

## Data Availability

The data underlying this article will be shared on reasonable request to the corresponding author.

## Abbreviations

HCD: Hypothermia-induced cardiac dysfunction
AP: Action potential
VF: Ventricular fibrillation
HCD: Hypothermia-induced cardiac dysfunction
TPR: Total peripheral resistance
CO: Cardiac output
PDE3: Phosphodiesterase 3
hiPSC-CM: Human-induced pluripotent stem-cell derived cardiomyocytes
PMT: Photomultiplier tube
APD30: Action potential duration 30%
APD50: Action potential duration 50%
APD90: Action potential duration 90%
Trise: Upstroke of the action potential
CtAmp: Contraction amplitude
CtD50: Contractility duration at 50% amplitude
CtTrise: Contraction rise time
S.E.M.: Standard error of mean
DMSO: Dimethyl sulfoxide
